# Potassium Iodide-Functionalized Polyaniline Nanothin Film Chemiresistor for Ultrasensitive Ozone Gas Sensing

**DOI:** 10.3390/polym9030080

**Published:** 2017-02-24

**Authors:** Sira Srinives, Tapan Sarkar, Raul Hernandez, Ashok Mulchandani

**Affiliations:** 1 Department of Chemical and Environmental Engineering, University of California-Riverside, Riverside, CA 92521, USA; sira.sri@mahidol.ac.th (S.S.); sarkar.ucr@gmail.com (T.S.); ruly.hernandez@gmail.com (R.H.); 2 Materials Science and Engineering Program, University of California-Riverside, Riverside, CA 92521, USA; 3 Department of Chemical Engineering, Mahidol University, 25/25 Puttamonthon 4 Rd., Nakorn Pathom 73170, Thailand; 4 University School of Chemical Technology, Guru Gobind Singh Indraprastha University, Sector 16C, Dwarka, New Delhi 110078, India

**Keywords:** ozone sensor, iodometry, polyaniline, gas sensor, potassium iodide

## Abstract

Polyaniline (PANI) nanostructures have been widely studied for their sensitivity to atmospheric pollutants at ambient conditions. We recently showed an effective way to electropolymerize a PANI nanothin film on prefabricated microelectrodes, and demonstrated its remarkable sensing performance to be comparable to that of a one-dimensional nanostructure, such as PANI nanowires. In this work, we report further progress in the application of the PANI nanothin film chemiresistive sensor for the detection of ozone (O_3_) by modifying the film with potassium iodide (KI). The KI-PANI sensor exhibited an excellent sensitivity to O_3_ (8–180 ppb O_3_ concentration rage) with a limit of detection of 230 ppt O_3_, and exquisite selectivity against active chemicals such as nitrogen dioxide (NO_2_) and sulfur dioxide (SO_2_). The sensing mechanism of the sensor relied on iodometric chemistry of KI and O_3_, producing triiodide ($I_{3}^{-}$) that partially doped and increased electrical conductivity of the PANI film. The sensitivity and selectivity of the KI-functionalized PANI film demonstrates the potential use for KI-PANI-based O_3_ sensing devices in environmental monitoring and occupational safety.

## 1. Introduction

Ozone (O_3_) is an oxidative tri-oxygen compound that exists at ppb-to-sub-ppm levels in the tropospheric atmosphere. Even though it is present at low concentrations, O_3_ is classified as a dangerous pollutant because it is poisonous to human health (Occupational Safety and Health Administration (OSHA) permissible exposure limit (PEL) of 100 ppb), harmful to crops, and involved in various atmospheric chemistries. The involvement of O_3_ in photochemical smog creates a dynamic equilibrium of O_3_ with other toxic pollutants, including nitrogen dioxide (NO_2_), nitric oxide (NO), and some volatile organic species. Moreover, O_3_ is a known greenhouse gas that contributes to global climate change. The serious impacts of O_3_ on atmospheric quality, biological/ecological systems, and personal health provokes public awareness regarding the detection and monitoring of atmospheric O_3_.

Various approaches are currently employed to determine atmospheric O_3_, including iodometric chemistry [1], spectrophotometry [2], and mass sensitive resonance [3]. These methods require experienced operators, elaborate and expensive lab equipment, and are not suitable for field measurement or personal safety purposes. Electrochemical approaches such as amperometry and potentiometry have also been reported [4]. The main concern with the electrochemical approaches is the low O_3_ solubility in liquid electrolyte, which limits the sensor sensitivity. In addition, common reactive species like NO_2_ and SO_2_ interfere with this measurement [5].

Considerations into creating miniature and chemiresistive sensors that could be employed in a portable device or a compact area led to a new class of sensors for air pollutants. The next generation approach to O_3_ detection emerged in sensor design using a combination of microfabricated electrodes and nanostructured material. Several types of material have been demonstrated to be sensitive to O_3_, including metal oxide [6], phthalocyanine [7], and carbon nanotubes [8]. The former two require elevated temperature to allow effective measurement, leading to high-energy consumption, while the latter suffers from a lack of selectivity. 

Conducting polymers such as polypyrrole (PPy), polyaniline (PANI) and polythiophene (PEDOT) have been shown as effective sensing elements in chemiresistive sensors. PANI is one of the primary foci in such sensors because of its good stability in ambient conditions, ease of processing, and wide range of tunable conductivity. The conductivity of PANI depends strongly on its oxidative state, which allows this polymer to react with species such as NH_3_, NO_2_, and H_2_. We previously reported the synthesis and characterization of nanothin film of PANI and its use for the detection of gases such as NO_2_, NH_3_, formaldehyde, and CO_2_ [9,10,11]. The nanothin film sensor showed outstanding sensing performance that was comparable to that of the one-dimensional nanostructure and offered advantages in fabrication simplicity and post-synthesis functionalization [9,11]. Different functional group, recognition layer, or metal catalyst could be integrated to the nanothin film via simple techniques, such as electroplating [12], and dip coating [9,11]. In this work, potassium iodide (KI) was incorporated in the PANI nanothin film via the simple dip coating method, and served as recognition site for iodometric reaction with O_3_ gas in the presence of water. Iodine (I_2_) and iodide (I^−^) obtained during the process subsequently reacted to form triiodide ($I_{3}^{-}$), that then partially doped PANI and modulated its conductivity. Cross reactivity of the KI-PANI sensor device was studied against NO_2_ and SO_2_. The effects of humidity and temperature on the sensor in relation to O_3_ detection were also investigated.

## 2. Materials and Methods

All chemicals were reagent grade, and used as received. Electropolymerization of PANI nanothin film was performed with standard three-electrode electrochemical cell via a CHI760C electrochemistry work station. The prefabricated gold microelectrode was used as substrate for the electropolymerization.

### 2.1. Sensor Fabrication

The microelectrodes were fabricated on highly-doped silicon substrate as pairs of 100 μm × 200 μm gold contacts, separated by 3 μm gap channel using photolithography. Prior to use, the microelectrodes were cleaned in a 3:7 (*v*:*v*) solution of hydrogen peroxide and sulfuric acid, rinsed with DI water, and blown dry under a nitrogen stream. The cleaned substrate with microelectrodes was further heated at 100 °C for 1 h and immediately submerged in a solution of 2% (*v*/*v*) octadecyltrichlorosilane (OTS) in toluene (anhydrous) to deposit an OTS self-assembled monolayer. The OTS-silanized silicon dioxide surface became highly hydrophobic after the silanization treatment, and attracted aniline monomer to accumulate on. The hydrophobic silicon dioxide surface helped to promote lateral film growth during electropolymerization [10,11], and was a key to realizing a nanothin film structure.

The microelectrodes acting as working electrode, Ag/AgCl reference electrode, and platinum mesh counter electrode were employed in the electrochemical cell in an electrolyte of 0.5 M aniline and 1 M perchloric acid (HClO_4_). Constant potentials of 0.6 and 0.8 V were applied individually to each pair of gold electrodes, and were terminated as soon as the chronocurrent curves (electrochemical current versus time) bifurcated from one another. The bifurcation indicated a complete film connection across the electrode channel as a result of ohmic current flow. The PANI nanothin film was 10–20 nm in thickness, with a highly porous surface structure [10]. More details on the film surface morphology and electrical characteristics of the nanothin film were previously shown by our group [10]. The film device was bathed in a 5 mM KI (pH ~ 7) aqueous solution for 15 min, during which the KI adsorbed and incorporated in the polymer matrix. The KI-functionalized PANI nanothin film was dried overnight in a low-vacuum desiccant for a future use.

### 2.2. Gas Sensing

Gas sensing experiments were performed inside a temperature-controlled gas sensing apparatus. All the sensor devices were sealed inside a 13 cm^3^ glass dome, and were poised at constant potential of 0.5 V. Electrical resistances of the sensor were acquired (chemiresistive mode) via a multichannel field point system, and were recorded as a function of operating time. All mass flow controlled streams were combined and mixed together in a tubular static mixer and delivered at a total flow rate of 100 cm^3^/min. Gas sensing parameters, including gas flow rate, time of analyte exposure, and time of the response recovery were assigned via the LABVIEW^®^ program (National Instruments, Austin, TX, USA), installed on a personal computer. O_3_ gas was generated from dry synthetic air (99% purity, Airgas Inc., Long Beach, CA, USA) using an OREC ozone generator (OSMONICS, SP series). The amount of O_3_ produced from the generator was pre-calibrated via iodometric procedures [1]. The generated O_3_ stream was diluted in synthetic air and immediately introduced to the sensor devices. The NO_2_ and SO_2_ were introduced from two separate certified gas cylinders (Airgas, Long Beach, CA, USA, 10 ppm).

All PANI film sensors were stabilized under continuous flow of air at a desired temperature and relative humidity before being exposed to analyte pulses. Each pulse included 15 min of analyte exposure followed by 35 min of analyte-free purging period. The windows of analyte concentration were fixed judiciously in this experiment considering suggested values in the national ambient air quality standard (NAAQS) that were regulated by the USA environmental protection agency (EPA). The values were 75, 100, and 75 ppb for O_3_, NO_2_, and SO_2_, respectively. 

## 3. Results and Discussion

### 3.1. Sensing with Pristine PANI Nanothin Film

As shown in our previous works [9,10,11], electropolymerization of PANI, performed by poising the two gold electrodes at 0.6 and 0.8 V vs. Ag/AgCl, produced a uniform 10–20 nm-thick carpet-like morphology PANI film of high surface area bridging the 3-µm gap channel between the gold electrodes (figure not shown). 

**Figure 1:**
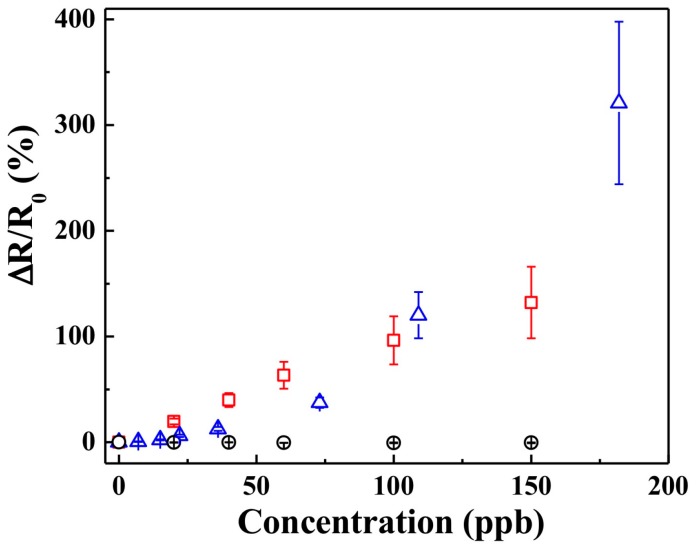
Normalized responses from the pristine polyaniline (PANI) sensors: (red/**□**) NO_2_, (blue/**∆**) O_3_, and (black/**○**) SO_2_; 25 °C; 50% relative humidity (RH). Data points are average of responses from three independent sensors, and error bars represent ±1 standard deviation.

Next, we evaluated the sensing performance of the pristine PANI sensor to gas pulses of NO_2_, SO_2_, or O_3_. The sensing results are presented as a calibration plot of normalized resistance change (Δ*R*/*R*_0_ (%)) versus analyte concentration (Figure 1). As illustrated in the figure, pristine PANI film-based sensors yielded no significant response to SO_2_, which is a weak reducing agent. On the other hand, strong oxidizing agents O_3_ and NO_2_ produced a significant increase in electrical resistance of the sensors, attributed to degradation of the PANI polymeric chain [13,14]. Further, while the sensing response from the pristine PANI nanothin film to O_3_ gas was strong, there was a significant cross reactivity from NO_2_. This interference would indeed be a problem in the detection of atmospheric ozone.

### 3.2. Sensing with KI-PANI Nanothin Film

**Figure 2:**
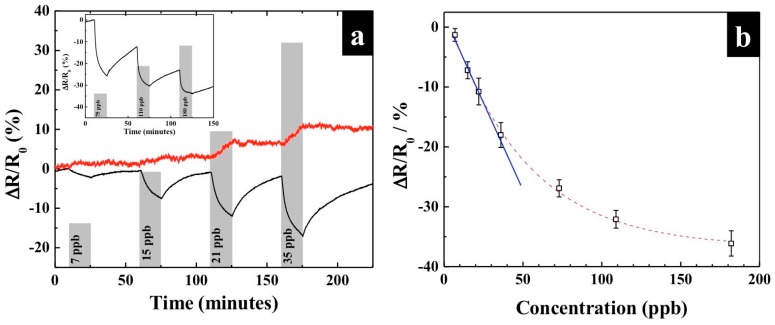
(**a**) Dynamic responses (Δ*R*/*R*_0_) of KI-functionalized PANI nanothin film (KI-PANI, black) and pristine PANI (red) sensors to 7–35 ppb of ozone gas at 25 °C and 50% RH. The inset shows sensing response of 75–180 ppb of ozone gas; (**b**) Calibration curve of KI-PANI sensor to 7–180 ppb of ozone gas at 25 °C and 50% RH.

To alleviate NO_2_ interference, we investigated the incorporation of iodometry in PANI-chemiresistor. The PANI film was modified with KI and employed for gas sensing at 25 °C and 50% relative humidity (RH). As shown in Figure 2a, KI-PANI devices exhibited a remarkable reduction in electrical resistance upon exposure to 7–180 ppb of O_3_ gas pulses. The KI-PANI sensor showed a wide concentration range that was linear up to 35 ppb (blue trace), and a linear regression equation of *Y* = −0.566*X* + 2 was obtained (Figure 2b). The sensitivity—slope of the regression equation—of ~0.566%/ppb O_3_ surpassed the sensitivity of sensors based on metal oxide (~1.50 × 10^−4^%/ppb O_3_) [6] and single-walled carbon nanotubes (~0.23%/ppb O_3_) [8]. In addition, the recovery of sensing response was very good during the analyte-free purging period after exposure to less than 40 ppb O_3_. An incomplete recovery at higher O_3_ concentrations may be ascribed to permanent oxidation of PANI. The limit of detection (LOD)—the lowest concentration of the substance that the device can distinguish from a blank sample with 99% confidence (calculated by LOD = 3 SD/s, where SD is the standard deviation of the noise and s is the slope of the linear part of the calibration curve)—was determined to be 230 ppt of O_3_.

The mechanism of sensing in the KI-PANI film sensor is based on the iodometric chemistry between KI and O_3_. In the presence of water, iodide (I^−^) from KI reacts with O_3_, yielding iodine (I_2_) as a reactive species (Equation (1)). The I_2_ then reacts with iodide (I^−^), based on the Lewis acid–base reaction, and converts into triiodide $\left(I_{3}^{-}\right)$ (Equation (2)). The $I_{3}^{-}$ further acts as dopant to the PANI, causing an increase in the film conductivity [14,15], as observed in sensing responses (Figure 2). However, the formation of $I_{3}^{-}$ is an endergonic reaction (∆*G* > 0) which is reversed in the absence of O_3_ due to the equilibrium shift upon ceasing of I_2_ production. This leads to a response recovery in the O_3_-free environment. It is worth mentioning that Stergiou and his team [16] reported a pH increment in an unbuffered solution of KI upon exposure to O_3_, as a result of hydroxyl (OH^−^) production. This phenomenon was not observed in our experiment due to a dominating effect from $I_{3}^{-}$ doping.

(1) $${2I}^{-}{+O}_{3}{+2H}^{+}↔I_{2}{+H}_{2}{O+O}_{2}$$

(2) $$I_{2}{+I}^{-}↔I_{3}^{-}\;$$

### 3.3. Effect of Humidity and Temperature

Humidity is an important factor for the sensor performance, since atmospheric humidity controlled the amount of water content in the PANI film. A sufficient amount of water was needed to avoid self-passivation of KI due to a formation of KIO_3_ [17,18]. Water dependency of the KI-O_3_ reaction led to an investigation of humidity and temperature effects on the sensing performance. 

**Figure 3:**
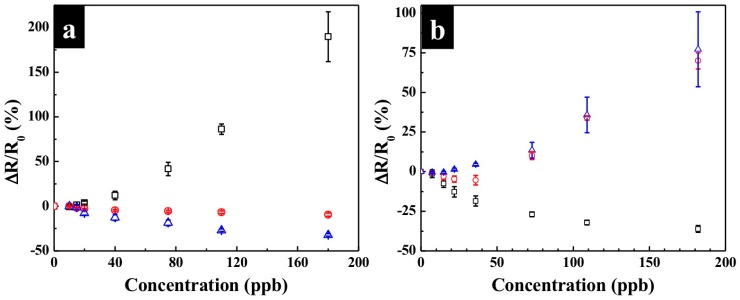
(**a**) Effect of humidity on the detection of O_3_ by KI-PANI device at 25 °C and 10% RH (black/**□**), 35% RH (red/**○**), and 50% RH (blue/**∆**); (**b**) Effect of temperature on the detection of O_3_ by KI-PANI device at 50% RH and 25 °C (black/**□**), 35 °C (red/**○**), and 50 °C (blue/**∆**). Data points are average of responses from three or more independent sensors, and error bars represent ±1 standard deviation.

The KI-PANI devices were fabricated and stabilized under fixed temperature and relative humidity. The results showed that response intensity of the KI-PANI device to O_3_ gas decreased as the RH decreased from 50% to 35% and 10% (Figure 3a). At 10% RH, the KI-PANI sensors responded to O_3_ pulses in positive/opposite direction; i.e., the resistance increased, revealing a domination of chemical oxidation reaction between PANI film and O_3_ over the iodometric reaction. On the other hand, temperature increase caused a reduction in the water content in the PANI film, and lowered the sensing performance of the sensors (Figure 3b) as a result of the self-passivating effect on the KI surface. This could also be attributed to the exothermic nature of the KI-O_3_ (acid–base) reaction.

While the effects of humidity and temperature on the sensor response may seem to be a problem, they can be solved by controlling the air sample humidity by humidifying/dehumidifying and establishing calibration plots at different temperatures for the determination of O_3_ concentration.

### 3.4. Cross-Reactivity

**Figure 4:**
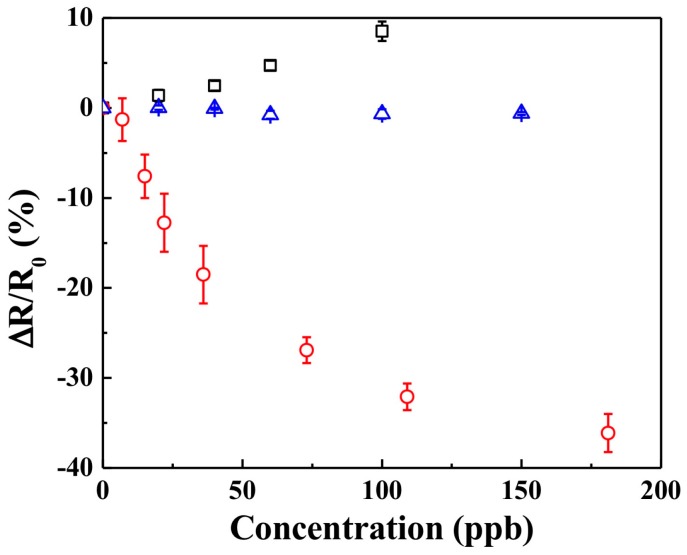
Selectivity of KI-PANI sensor towards O_3_ (red/**○**) with respect to other interfering analytes NO_2_ (black/**□**) and SO_2_ (blue/**∆**) in terms of sensing response (**∆***R*/*R*_0_) at various analyte concentrations. Data points are average of responses from three independent sensors, and error bars represent ±1 standard deviation.

Since the KI-PANI sensor device relies on iodometric chemistry, it may be susceptible to interference from reactive gases such as NO_2_ and SO_2_. To investigate this, the response of the KI-PANI device to NO_2_ and SO_2_ pulses was studied at 25 °C and 50% RH. A calibration plot (presented in Figure 4) showed no significant response of the KI-PANI device to SO_2_ gas, and only a slight response to NO_2_ above 25 ppb. This response, however, was opposite to that for O_3_; i.e., the resistance increased instead of decreased. The sensitivity and LOD were ~0.08%/ppb NO_2_ and ~2.5 ppb NO_2_, respectively. The sensitivity observed in this case was significantly lower than that for O_3_ on KI-PANI and also for NO_2_ on pristine PANI (Figure 1). A slight increase in the resistance of the device to NO_2_ suggested that the oxidation reaction (PANI-NO_2_)—which yielded resistance increase—was more predominant than the iodometric reaction (KI-NO_2_), which caused a resistance decrease due to partial doping of PANI with $I_{3}^{-}$. 

**Figure 5:**
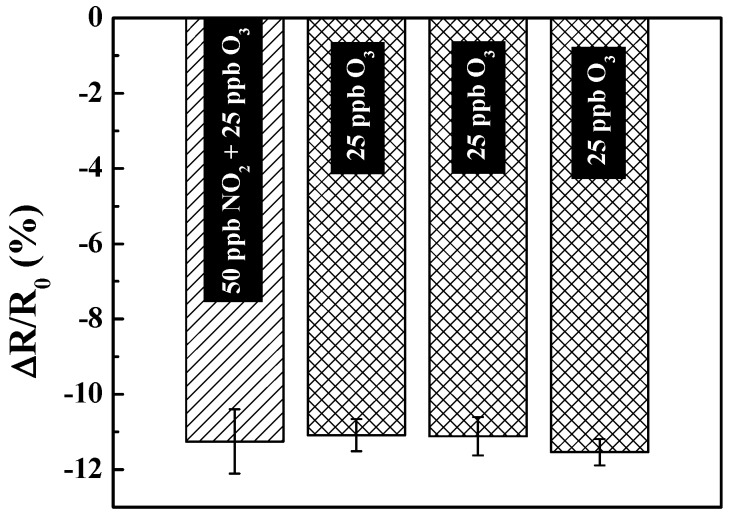
Response (**∆***R*/*R*_0_) of KI-PANI device towards pure O_3_ (25 ppb) and mixed gas [O_3_ (25 ppb) + NO_2_ (50 ppb)] at 25 °C and 50% RH. Data points are average of responses from three independent sensors, and error bars represent ±1 standard deviation.

To assess the interference from NO_2_ when present together with O_3_ in an analyte sample, the response of KI-PANI sensor to a mixture of 25 ppb O_3_ and 50 ppb NO_2_ was compared to that for 25 ppb O_3_ only. The result showed that there was an insignificant difference in the response of the sensor between the two samples (Figure 5). This is attributed to a significantly high thermodynamic preference of KI in reacting with O_3_ to NO_2_ gas as evidenced by the very high standard potential, *E*°, for KI-O_3_ reaction (*E*° = 1.409 V) over that of the KI-NO_2_ reaction (*E*° = 0.179) [19,20]. As an alternative, to completely eliminate NO_2_ interference, air could be filtered through a packed bed of potassium permanganate (KMnO_4_) to eliminate NO_2_ [7]. In the same manner, manganese dioxide (MnO_2_) [7] can be used to take out the O_3_, and provide the O_3_-free air stream for the sensor purging purposes. Filtering/treating the air/gas sample by KMnO_4_ and MnO_2_ would also help to alleviate any baseline drift resulting from the chemical oxidation of PANI. Additionally, the calculated ozone concentration (using the regression equation fitted to the linear region of the calibration plot in Figure 2b) of 23.41 ± 3.89 ppb was in excellent agreement with the actual concentration of 25 ppb used in the experiment; a 93.64% relative accuracy. Further, the excellent reproducible response to successive detection of ozone (last three histograms in Figure 5) demonstrates the excellent stability of the sensors.

## 4. Conclusions

An O_3_ sensing device was fabricated here, based on a KI-functionalized PANI nanothin film. The KI-PANI film exhibited a remarkable sensing performance toward 8–180 ppb of O_3_ analyte, with the LOD value of 230 ppt, surpassing other sensitive elements such as carbon nanotubes [8] and metal oxide [6]. The sensing mechanism relied on the iodometric reaction of KI and O_3_ in the presence of water that formed iodine, iodide, and triiodide. The triiodide can dope and increase the conductivity of the PANI film. The sensor also showed discrepancy against interferences, including NO_2_ and SO_2_.
